# Non-invasive assessment of vascular alteration using ultrasound

**DOI:** 10.1186/s40885-015-0035-4

**Published:** 2015-12-16

**Authors:** Woo-In Yang, Jong-Won Ha

**Affiliations:** Cardiology Division, CHA Bundang Medical Center, CHA University, Sungnam, Republic of Korea; Cardiology Division, Severance Cardiovascular Hospital, Yonsei University College of Medicine, Seoul, Republic of Korea; Severance Biomedical Science Institute, Yonsei University College of Medicine, 134 Shinchon-dong, Seodaemun-gu Seoul, 120-752 Republic of Korea

**Keywords:** Arterial function, Carotid artery, Ultrasound

## Abstract

It is well known that arterial function relates to cardiovascular morbidity and mortality. The noninvasive technology for the assessment of arterial function has developed as the importance of prevention of early cardiovascular disease has been emphasized. Over 2-D and Doppler echocardiography, speckle-tracking echocardiography has emerged as a valuable ultrasound imaging technique that allows for an objective and quantitative evaluation of global and regional myocardial function. Recently, several studies have extended its applicability beyond cardiac chambers, such as artery. Measurement of carotid arterial strain with speckle tracking ultrasound has been shown to be feasible and reliable. This review describes the new ultrasound techniques to assess arterial function and their clinical implications.

## Background

### Why do we need to evaluate arterial function?

Cardiovascular disease (CVD) is the leading cause of morbidity and mortality in the industrialized countries [1]. In the early half of the 20th century, CVD increased rapidly as a result of industrialization and urbanization. However, during the latter half of the 20th century, the mortality of CVD decreased impressively due to advances in medical technology [1]. Coronary angiography has been the standard for the diagnosis and management of coronary artery disease for decades. However, physician’s interests are going over to noninvasive assessment of vascular function as the importance of prevention of early CVD has been emphasized.

Previously, vascular aging, the changes of vascular function and structure, was overlooked in comparison with overt atherosclerosis. The cardiovascular continnum was restricted to atherosclerotic disease [2, 3]. However, vascular aging, in association with increased arterial stiffness, influences on the heart and other organs [4]. Vascular aging is correlated with systolic hypertension, atherosclerosis, diastolic heart failure and small vessel disease in brain and kidney [4]. It is well known that arterial function relates to cardiovascular morbidity and mortality [5–7]. In the late 19th century, William Osler already underscored the importance of vascular aging by saying “you are as old as your arteries.” Recently vascular aging has become one of the most important issue and regarded as the extended cardiovascular continnum [8]. These changes engage with the development of noninvasive technology for the assessment of arterial function.

This review describes the new ultrasound techniques to assess arterial function and their clinical implications.

## Basic pathophysiology of arterial function

The arterial wall is mainly composed of scaffolding proteins, elastin and collagen [9]. Repetitive pulsation (approximately 30 million per year) results in damage of elastic lamellae and replacement with collagen in the arterial walls [10]. In various vasculopathy or with aging, elastin becomes broken and diminished in quantity [10]. Furthermore, the collagen matrix increases in a disorganized form [10]. These changes result in arterial dilatation and stiffening [4]. As arterial stiffness increases, flow pulsations cannot be buffered and are directly transmitted to distal arteries. This repeated mechanical stress damages arterial wall and results in vascular inflammation and atherosclerosis [11–13]. Generally, arterial pressure wave analysis and pulse wave velocity (PWV) have been regarded as valuable tools to evaluate arterial stiffness [14, 15]. However, these methods assess global vascular changes rather than local alterations, which usually precede. Therefore, to detect early changes of vascular alteration, techniques that can evaluate local vascular changes would be quite useful, such as vascular ultrasound. Among the numerous arteries, common carotid artery is most frequently used for the assessment of arterial properties since it allows good spatial resolution when using ultrasound. During the arterial assessment, various parameters can be measured and carotid intima-media thickness (cIMT) has been suggested as a surrogate measure of vascular alterations, and several studies have shown that increasing cIMT is associated with subsequent coronary heart disease and stroke [16, 17].

Since it is non-invasive, reproducible, and simple, measurement of cIMT with ultrasonography is widely used to quantify the extent of subclinical disease in individuals with cardiovascular (CV) risk factors and to follow up in interventional studies. However, cIMT may not reflect the whole arterial changes occurring in various pathologic conditions. Before the progression of the intima and media thickening, the carotid artery may experience functional alterations, of which the occurrence is also associated with an increased risk of CV morbidity or mortality by augmenting arterial impedance and resultant increase in the afterload of the heart [18]. Recently, with advancements in ultrasound techniques, a novel technique has facilitated the instantaneous quantification of carotid arterial mechanics.

## Novel technique in evaluation of vascular function; strain imaging

Over 2-D and Doppler echocardiography, speckle-tracking echocardiography has emerged as a valuable ultrasound imaging technique that allows for an objective and quantitative evaluation of global and regional myocardial function [19, 20]. Speckle-tracking echocardiography analyzes the spatial dislocation of speckles (defined as spots generated by the interaction between the ultrasound beam and myocardial fibers) on routine 2-D ultrasound imaging [21]. By tracking the displacement of speckles during the cardiac cycle, speckle-tracking echocardiography evaluates myocardial deformation. The 2-D speckle tracking method allows for angle-independent, rapid, and direct assessment of tissue motion and deformation [19, 20]. Based on this new technique, greater understanding into the pathophysiology of various cardiovascular diseases could be gained. Recently, several studies have extended its applicability beyond cardiac chambers, such as artery.

Measurement of carotid arterial strain with speckle tracking ultrasound has been shown to be feasible and reliable [5]. Recent studies reported that circumferential strain of common carotid artery was more sensitive in discrimination of aged artery and risk stratification, compared with cIMT and conventional 2-D ultrasound derived stiffness variables [22, 23]. In a study with coronary artery disease, carotid arterial strain was related with the presence and extent of coronary artery disease [24]. Svedlund et al. demonstrated that reduced longitudinal function of common carotid artery assessed by strain imaging predicted adverse cardiovascular event after 1 year [25].

**Fig. 1:**
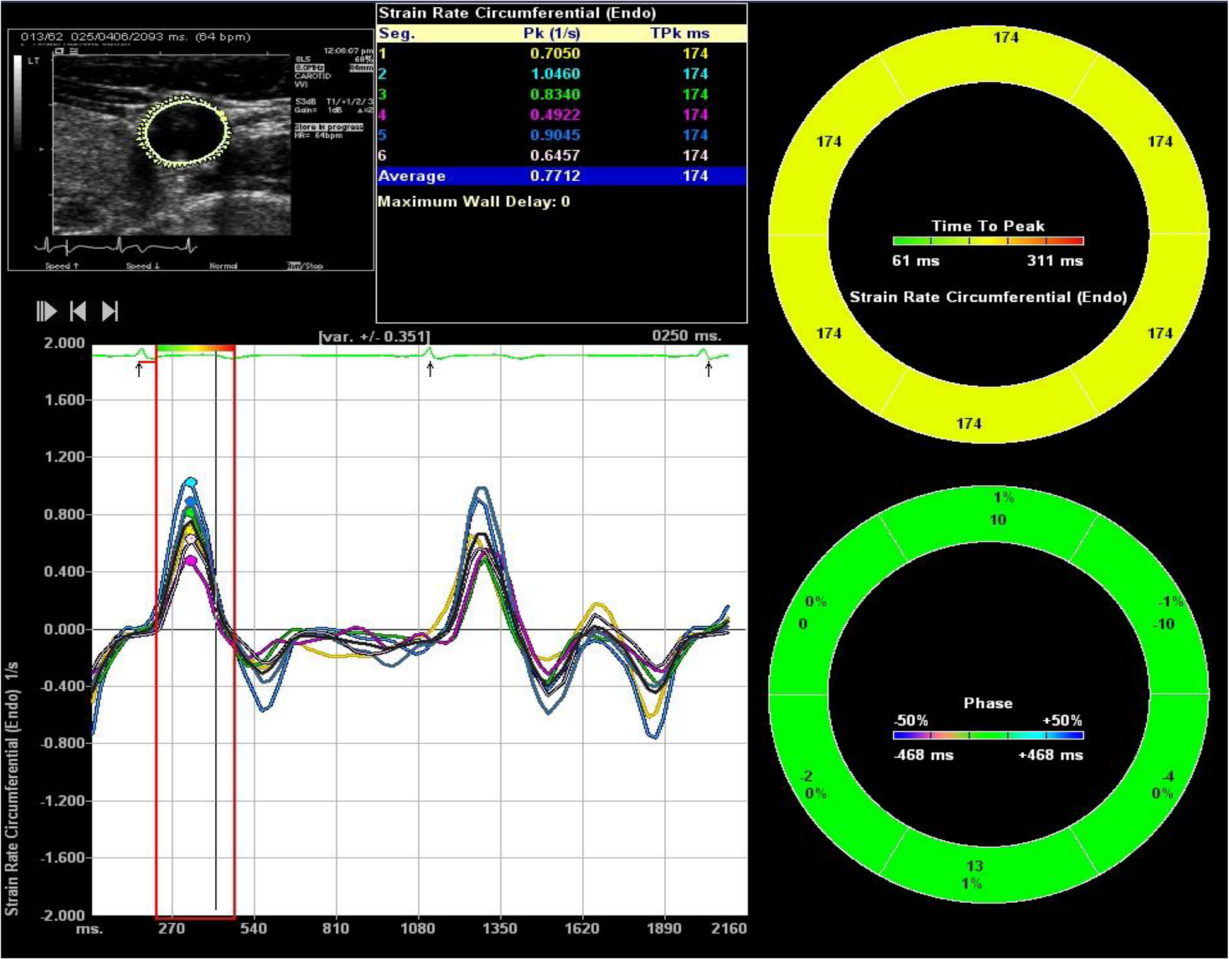
The evaluation of arterial wall mechanics using VVI: The media-adventitia borders of carotid arteries were manually traced, and the peak circumferential strain rate and times to peak circumferential strain rate of six segments were analyzed automatically. The peak circumferential strain and the times to peak strain were similarly measured

**Fig. 2:**
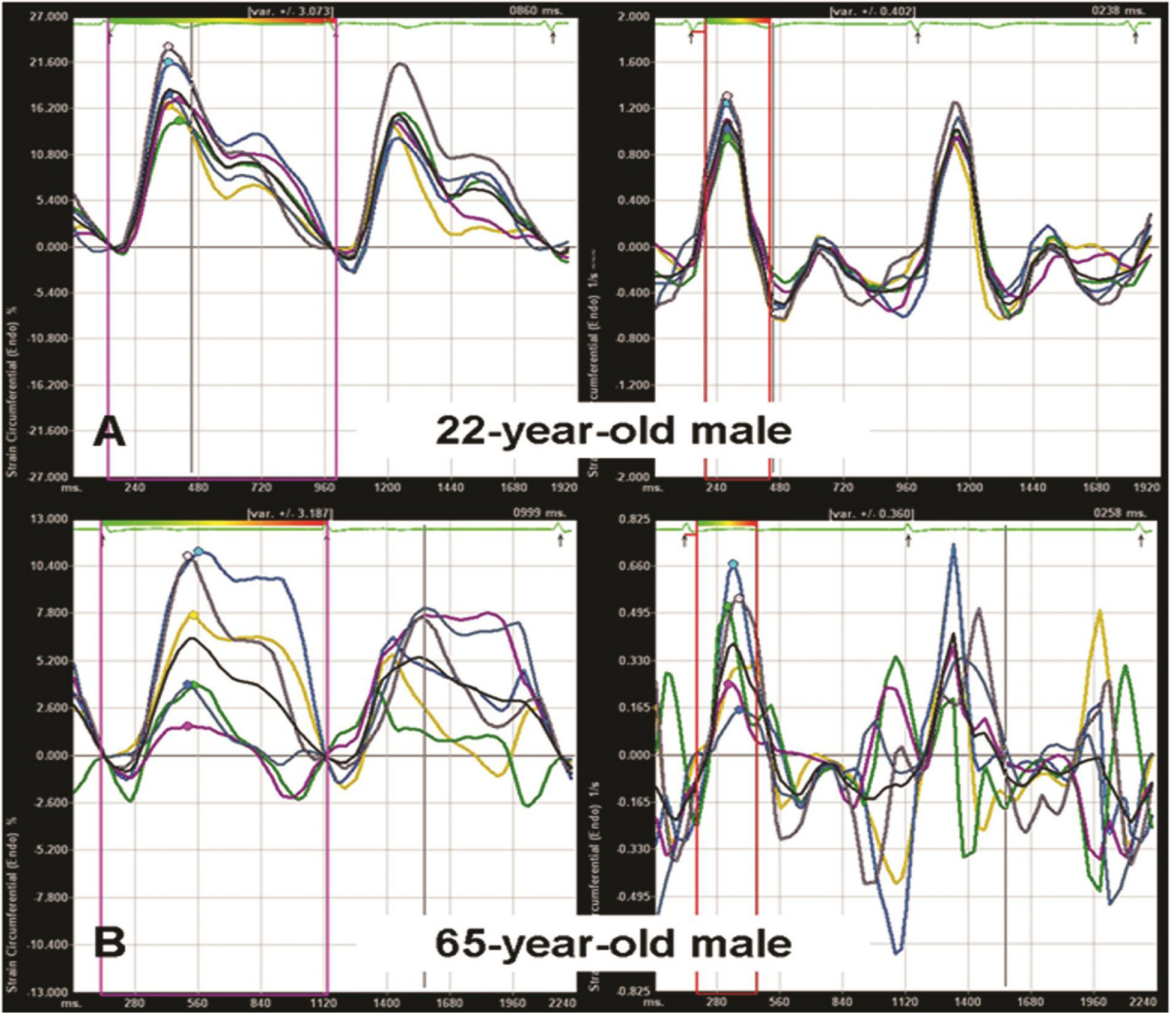
VVI analysis: circumferential strain and strain rate of six segments in a 22-year-old male subject (**a**) and a 65-year-old male subject (**b**). The carotid artery of the older subject showed a more reduced and asynchronous arterial expansion during systole. VVI = velocity vector imaging (adapted from Ref 29)

**Fig. 3:**
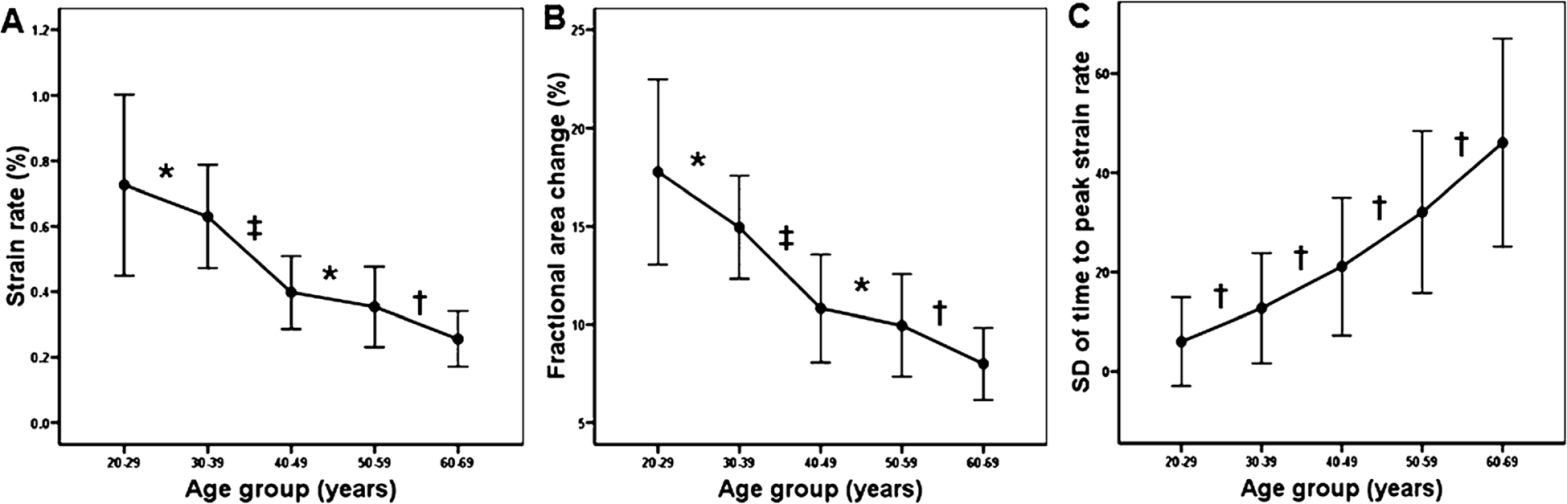
**a** Average peak circumferential strain rate according to age group. **b** Fractional area change according to age group. **c** The SD of time to peak circumferential strain rate (Tsr-SD) according to age group. Data are presented as mean ± SD. SD = standard deviation; Tsr = time to peak circumferential strain rate. *P > 0.05, †*P* < 0.05, ‡*P* < 0.01(adapted from Ref [29])

Velocity vector imaging (VVI) is a new method of strain imaging with 2-D speckle tracking [26, 27]. We have previously shown the feasibility of using VVI in the evaluation of arterial wall mechanics and have demonstrated the characteristics of arterial mechanics accompanying aging and vasculopathies using VVI (Fig. 1) [28–30]. Fractional area change and strain and strain rate by VVI were reduced according to age and significantly correlated with conventional parameters of arterial stiffness such as PWV and augmentation index (AIx) [29]. More importantly, VVI measurements of time to peak strain and strain rate enabled the assessment of the synchronicity of arterial systolic expansion. The SD of time to peak strain and strain rate significantly increased with age, suggesting non-uniform arterial expansion during systole with age (Fig. 2). PWV and AIx are considered as representative markers for vascular aging. However, PWV is increased more prominently in older age whereas AIx is a sensitive marker of arterial stiffening in relatively younger subjects [15]. Unlike PWV or AIx, asynchronicity of arterial systolic expansion rises steadily with aging and is independent of hemodynamic conditions including heart rate (Figs. 2 and 3) [29]. Therefore, asynchronous arterial expansion could be a more sensitive marker of vascular aging at all ages.

Arterial function using VVI was also evaluated in patients with vasculopathy such as Takayasu’s arteritis and Marfan syndrome [28, 30]. In Takayasu’s arteritis and Marfan syndrome, arterial strain and strain rate were all reduced. Furthermore, SD of time to peak strain and strain rate were significantly increased, suggesting dyssynchronous arterial expansion during systole. According to the previous investigations, VVI has unique ability to provide an information regarding both regional and segmental alterations of arterial elastic properties in various conditions. In an animal study to assess the correlations between the VVI parameters and histologic changes, the radial velocity and circumferential strain had independent associations with the collagen content of the corresponding aortic wall [31].

Previously, most studies on arterial aging have focused on global changes of arterial function due to lack of test for regional arterial function. Conventional methods for assessment of arterial function usually represent global arterial function, and not regional function. Despite its potential importance, regional structural changes of artery and consequent aynchronicity of arterial expansion has been overlooked. Unlike other assessment technique for arterial function, strain analyses present regional arterial function. Therefore, assessment of vascular function with strain imaging could be expected to offer better understanding into the pathophysiology of vasculopathy.

Carotid 2-D ultrasound evaluates not only arterial structure but also arterial function including changes in diameter during the cardiac cycle [14]. However, these assessments are limited, in that they can be influenced by blood pressure and have less than optimal reproducibility [32, 33]. In the Rotterdam study, carotid distensibility as assessed by ultrasound failed to show any association with cardiovascular disease or mortality [6]. Even though there are not many studies, compared with ultrasonographic 2-D measures of arterial stiffness, arterial strain and strain rate are more sensitive for detecting age-related vascular changes and determining cardiovascular risk [22, 23, 34].

However, up to the present, there are some limitations in evaluation of the arterial function with speckle tracking echocardiography. In cases of deep carotid arteries or compression by surrounding structures such as an enlarged thyroid or extended jugular vein, tracing of the carotid artery is technically difficult. Arterial wall is very thin compared to myocardium, so could be difficult to track. Even though, intra and interobserver variability were reliable in small studies, further studies with large subjects are necessary. The superiority of strain analysis compared with other methods of vascular functional assessment has not been confirmed yet. Longitudinal follow-up study with large populations needs to be evaluated to support the clinical usefulness of arterial strain analysis.

## Conclusions

Recently, new ultrasound techniques have enabled the assessment of arterial mechanics using strain analysis. Several studies reported the feasibility of strain analysis in the evaluation of arterial function. The results of those studies demonstrated that strain analysis of superficial artery could be a valuable method to evaluate arterial function. If some technical problems in acquiring proper images and tracking thin arterial wall are improved, evaluation of arterial strain could be used extensively to assess local arterial function in future.

## Abbreviations

CVD: Cardiovascular disease
PWV: Pulse wave velocity
cIMT: Carotid intima-media thickness
CV: Cardiovascular
VVI: Velocity vector imaging
AIx: Augmentation index
